# Plant–Microbiome Crosstalk: Dawning from Composition and Assembly of Microbial Community to Improvement of Disease Resilience in Plants

**DOI:** 10.3390/ijms22136852

**Published:** 2021-06-25

**Authors:** Muhammad Noman, Temoor Ahmed, Usman Ijaz, Muhammad Shahid, Azizullah, Dayong Li, Irfan Manzoor, Fengming Song

**Affiliations:** 1 State Key Laboratory of Rice Biology and Ministry of Agriculture Key Laboratory of Molecular Biology of Crop Pathogens and Insects, Institute of Biotechnology, Zhejiang University, Hangzhou 310058, China; nomansiddique834@gmail.com (M.N.); temoorahmed@zju.edu.cn (T.A.); usmanijazahmad1246@gmail.com (U.I.); azizullahkeerio0706@yahoo.com (A.); dyli@zju.edu.cn (D.L.); 2 Department of Bioinformatics and Biotechnology, Government College University, Faisalabad 38000, Pakistan; mshahid@gcuf.edu.pk; 3 Department of Biology, Indiana University, Bloomington, IN 47405, USA; imanzoor@iu.edu or

**Keywords:** microbiome engineering, microbiota, pathogens, plant–microbe interactions, rhizosphere, root exudates

## Abstract

Plants host diverse but taxonomically structured communities of microorganisms, called microbiome, which colonize various parts of host plants. Plant-associated microbial communities have been shown to confer multiple beneficial advantages to their host plants, such as nutrient acquisition, growth promotion, pathogen resistance, and environmental stress tolerance. Systematic studies have provided new insights into the economically and ecologically important microbial communities as hubs of core microbiota and revealed their beneficial impacts on the host plants. Microbiome engineering, which can improve the functional capabilities of native microbial species under challenging agricultural ambiance, is an emerging biotechnological strategy to improve crop yield and resilience against variety of environmental constraints of both biotic and abiotic nature. This review highlights the importance of indigenous microbial communities in improving plant health under pathogen-induced stress. Moreover, the potential solutions leading towards commercialization of proficient bioformulations for sustainable and improved crop production are also described.

## 1. Introduction

Interest in the control of crop diseases has recently increased due to the global requirement for eco-friendly approaches that would replace chemical fertilizers and pesticides in agricultural practices [1,2]. Plants provide the place for the growth of niches and the proliferation of a diverse microbial community, including protists, fungi, bacteria, viruses, and nematodes [3,4]. These organisms play important roles in the health and productivity of crops by forming complex co-associations with plants [1]. In particular, plant-associated microbiota and plants form a ‘holobiont’, and evolutionary selection among microbes and plants contributes to the stability of ecosystem [5,6]. Complex plant–microbial associations have deep branching lineages and comprise of diverse phyla at lower phylogenetic resolutions. Recently developed culture-independent high-throughput sequencing has accelerated the identification of microbial communities inhabiting the surrounding spaces, as well as inside tissues and surfaces of plants, and demonstrated the existence of microbial lineage subsets, termed as ‘core microbiota’, which reproducibly make contacts with host plants across a wide range of environmental conditions [7,8]. Among the plant microbiome, fungi and bacteria, which play significant roles in the proper functioning and health of plants [9,10], are dominant microbes in contrast to the other members of community, i.e., archaea, nematodes, algae, and protists. The metagenome/genome-wide association studies (M/GWAS) have enlightened our understanding regarding the roles of individual taxa in modulating plant physiology, colonization and fitness [8], whereby, multi-omic approaches have enabled us to predict and characterize the genes that facilitate the microbes to interact with the plant-associated microbiomes [11]. Based on the current knowledge of plant–microbiome interactions, there is an evolving paradigm that considers plant–microbiome associations/interactions as means to develop novel plant genotypes under continuously changing ambiance [12,13]. This review summarizes the recent advances on plant–microbiome interactions at the community level, along with the roles of composition and assembly of a microbial community to improve the disease resilience in plants. Current knowledge gaps and future research directions are also discussed. 

## 2. Structural Dynamics of Microbiome in Plant Life

**Figure 1:**
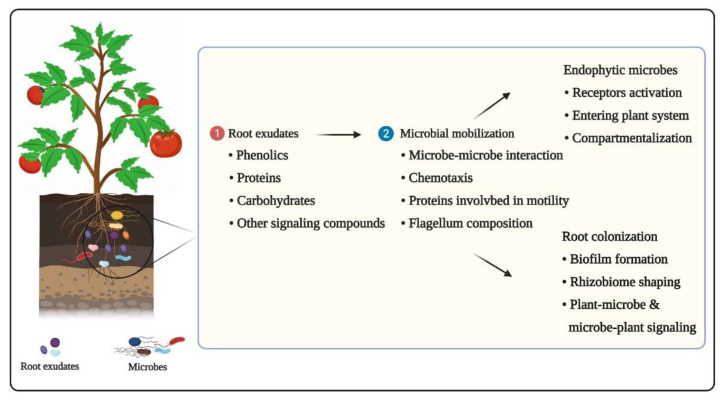
Various steps that occur during the assembly of the plant microbiome are depicted schematically. Firstly, plants release signaling molecules (i.e., phenolics, proteins, etc.) in the form of rhizodeposits to attract microbial community (step 1). Secondly, microbes respond to plant oriented signaling molecules by initiating mobilization and colonizing various plant parts as epiphytes and endophytes (step 2). The final diversity of the plant microbiome is shaped by a series of stages involving intricate signaling between plants and microorganisms.

The plant-associated microbiome assembly comprises of a series of successional steps determined by microbe–microbe and plant–microbe interplays (Figure 1). Plant-microbiota generally transmit via either horizontal pathway (i.e., obtained from the vicinal environment) or vertically (i.e., gained directly from the parent) [14]. Although the detailed information on horizontal and vertical modes of transmission remains elusive, their roles in shaping the final diversity of seed microbiota are well-established. Once seeds germinate, microbe assembly is stimulated by horizontal transmission mode, where seed-borne microorganisms mostly get fixed with roots and rhizosphere [15].

The microbiome associated with plant roots is dynamically assembled and recruited during life cycle of the host plants. Temporal alterations in structural composition of rhizobiome are constant across different geographic regions around the globe [16]. The microbiome composition is highly dynamic during early vegetative growth stage and sustains in later vegetative stage [16,17]. However, some members of microbial taxa belong to the core microbiota, and constantly maintain in relatively high abundances during the developmental phase of the host plants [16,17,18,19]. These core microbiota possess several beneficial traits, such as endurance of stress, beneficial effects on the host growth, efficient colonization, and to protect host plants from harsh environmental constraints [15,20]. Host plants, on the other hand, can transfer the abovementioned core microbiota-induced beneficial traits to their offspring, revealing the significance of linkage between plant-associated microbiome and host plants [21]. 

Plants harbor diverse microbial communities, some of which enter the plant tissues, called endophytes, while others stay on the outer surface of plant tissues, known as epiphytes [22,23]. Early studies mainly focused on the structural and functional characterization of rhizobiome, and discovered that plant–soil crosstalk plays a key role in shaping the rhizosphere microbiota [24]. For example, defects in phosphate starvation response (PSR) pathway in *Arabidopsis* plants negatively regulated the diversity of beneficial microbiota and ultimately favored the colonization of phytopathogens [25]. Plant leaves also offer habitats to complex and diverse microbial communities [26,27]. Most endophytes spread systematically through the xylem system to other compartments of plants such as the leaves, fruits, and stem; however, distinct endophyte communities are present on aboveground plant tissues depending on the plant source allocation [22]. Phyllosphere bacteria initially start their lives in a soil environment, and eventually enter into plant leaves as endophytes, a process driven mainly by environmental and plant factors [28,29,30]. Features of plant cell walls play key roles in shaping almost 40% of the bacterial population diversity in the roots of *Arabidopsis* plants [31]. Host genotype, age, and environment conditions have cumulative impacts on the diversity of rhizospheric and phyllospheric bacterial communities in *Boechera stricta*, suggesting the importance of genotype–environment interactions in determining the structural assembly of plant microbiomes under natural conditions [32].

Various microbial groups, belonging to different genus and species, inhabit the phyllosphere and endosphere of host plants. For instance, *Pseudomonas*, *Sphingomonas*, *Frigoribacterium*, *Pantoea*, *Acinetobacter*, *Enterobacter*, *Methylobacterium*, *Bacillus*, and *Curtobacterium* are predominant genera of carposphere or phyllosphere microbiota in grapevine [29,33], while *Methylobacteria* and *Sphingomonads* are predominant taxa of leaf microbiomes in maize [30]. Similarly, *Enterobacteriaceae* and *Pseudomonas* were identified as dominant epiphytic bacteria existing on the flower of apple [34], and *Pseudomonas* is the most abundant genus found in the leaves of tobacco, apple, pumpkin, grapefruit, and almond [35].

Plant endophytes mainly originate from seed, air, and soil, followed by habituation inside the plant tissues, where they spend rest of their lives. Various factors including environment factors, farm management, plant genotype, and soil features shape the community composition of plant endophytes [26,36]. Plants compartmentalize specific microbial communities as endophytes and establish a strong association as well as a signaling nexus with endophytes [37]. For example, invasion of *Xanthomonas oryzae* pv. *oryzae* (*Xoo*), the causal agent of rice bacterial blight, negatively regulated the endophytic microbial diversity of rice plants by reducing alpha-diversity of the fungal communities, *Xoo* infection helped rice plants to acquire disease combating beneficial microbes that subsequently elicited the disease-suppressive mechanisms in the plants [38]. However, the composition, interactions, and functions of endophytic bacterial communities in protecting plants from pathogen attack under adverse environmental conditions remain unclear. 

## 3. Plant–Microbe Interplays: Recruiting Microbial Communities for Microbiome Assembly

**Figure 2:**
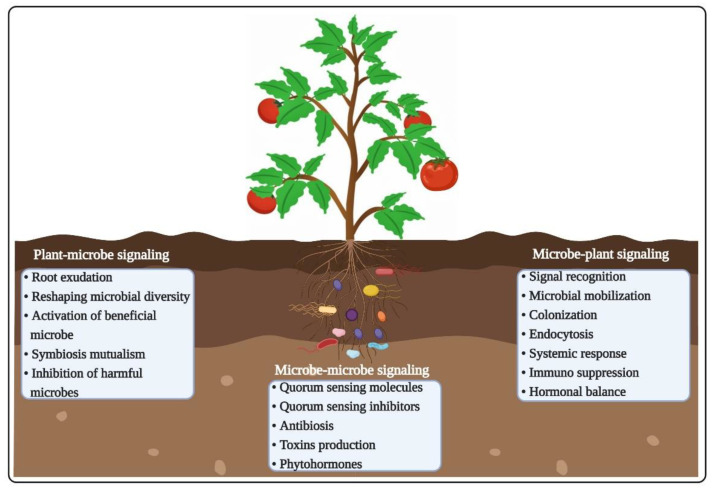
Schematic visualization of various interactions occurring in the plant holobiont. Numerous complex signaling pathways are involved in plant–microbiome crosstalk, including plant–microbe, microbe–microbe, and microbe–plant communications. The ultimate fate of plant–microbiome interactions depends on the chemistry of the rhizosphere, and the diversity and the composition of microbial communities.

Diverse microbial communities colonize plant surfaces and tissues, where beneficial microbial groups provide plants with a wide array of life supporting functions, such as resilience to biotic and abiotic stresses, growth promotion, and nutrient acquisition [39,40]. Managing microbial colonization process would help to modulate the abovementioned functions, but in-depth understanding regarding how plant genotypes regulate colonization of particular microbial group will be helpful to further strengthen beneficial microbiota-linked traits. The microbiome assembly depends on both plant–microbe interactions and microbe–microbe interactions (Figure 2). 

### 3.1. Root Exudates and Chemotaxis

Microbes employ chemotaxis to detect and respond to plant-derived signals (i.e., sugars or organic acids), exuded from plant roots, to initiate microbial colonization step. Following the signal perception, microbes mobilize towards plants and become attached to the surface of roots to form biofilm [41]. Genes responsible for motility, chemotaxis, biofilm formation, flagella assembly, two-component regulatory system, and secretions are abundantly present in microbial communities of phyllosphere and rhizosphere, in contrast to the bulk soil [42,43,44]. Large numbers of substrate transporters present in the members of phyla Firmicutes and Proteobacteria facilitate the habituation of microbial populations in the nutrient rich environment of plants [4,18,29]. Similarly, motility genes were also identified in bacterial strains isolated from *Arabidopsis thaliana* roots [45].

In plants, the compounds that stimulate chemotaxis in microbes are present on the root surface or in root exudates [46,47,48]. Detailed characterization of root exudates is challenging, owing to the variation in their composition with plant developmental stages, plant varieties, and environmental conditions [49]. However, several compounds have been identified in certain plant species; some are common, while others are unique [41]. Usually, polysaccharides are secreted by root tips and abundantly present in root caps and mucilage [50]. However, elongation zones and meristem contain oxidized compounds such as amino acids, sugars, and organic acids [51,52]. 

The ability to sense organic compounds widely exists in plant-beneficial bacteria including *Azospirillum brasilense*, *Sinorhizobium meliloti*, *Rhizobium leguminosarum*, and various *Pseudomonas* species, and specific receptors for different organic compounds have been identified in plant-beneficial bacteria [53]. *R. leguminosarum*, *A. brasilense*, and *S. meliloti* utilize organic acids as catabolite repressors [54]. Organic acids are the key metabolic regulators that help microbial species to adapt to rhizospheric environment, elucidating the widespread distribution of organic acid-mediated chemotaxis in plant-associated bacteria [55]. Microbes sense the molecules of interest present in root exudates indirectly, by periplasmic binding proteins and a phosphotransferase system followed by the attachment to corresponding receptors. For instance, a galactose binding periplasmic protein (i.e., ChvE) is involved in chemotaxis of *A. brasilense*, although the interacting chemoreceptors are still unknown [56]. *R. leguminosarum* and *S. meliloti* perform nitrogen fixation only during symbiotic association, and rely on other nitrogen sources (i.e., amino acids) under free-living conditions [54]. In addition, chemotaxis towards flavonoids, host-specific phenolic compounds exuded in low amount from plant roots, regulates the expression of nodulation genes in bacteria [57]. Some of the rhizodeposits secreted by different plants, together with microbial responses and effects, are presented in Table 1. 

Root colonization is generally triggered by Rhizobiales (part of the core microbiome) equipped with symbiosis-related genes [72]. In the interface of plant rhizospheres, polyamines, i.e., putrescine and arginine, act as signaling molecules and stimulate the lifestyle switch in a microbial group to promote attachment and biofilm formation [73,74]. After successful colonization within host plants, diverse processes, such as nutrient deficiency-mediated root inhibition and/or activation of signaling pathways, take place, which alter the root architecture of the host plants, resulting in differential niche colonization patterns between many microbial groups [75].

### 3.2. Microbe–Microbe Interactions

It is not surprising that various active genes of soil microbiota play significant roles in competition or cooperation with other microbes. Microbes can synthesize different products, which affect the microbe–microbe interactions. Distinct and diverse gene clusters for biosynthesis of natural products have been identified in plant-associated bacteria [76]. The genomes of plant-associated bacteria harbor biosynthetic genes for intraspecific or interspecific bacteria-killing substances (such as antibiotics and toxins) to control the abundance, diversity, and distribution of other microbial groups in host plants [55]. However, how these compounds are synthesized, and how they regulate the microbe–microbe interplays, along with their underlying biochemical mechanisms, remains to be explored.

Specific functional traits correlated with pathogen suppression, e.g., biosynthetic genes for antifungal compounds and protein secretion, are rich in bacterial disease combating rhizobiomes of tomato and soybean [77,78]. Pathogens induce the activation of various unknown biosynthetic gene clusters (BGCs), encoding polyketide synthases (PKSs), chitinases, and non-ribosomal peptide synthetases (NRPSs), which subsequently trigger the disease suppression activity in the endophytic root microbiome [79]. More than one thousand BGCs belonging to diverse biosynthetic classes of terpene system, post-translationally modified and ribosomally synthesized peptides, trans-AT PKSs, and NRPSs have been identified in phyllospheric bacteria of Arabidopsis plants [80]. These BCGs are involved in driving the complex interactions occurring within a microbial niche [81]. In addition to the abundance of antibiotic production, antibiotic resistance genes are also widely distributed within the microbiome [6], implying an intense competition between different microbial groups that controls the structure of microbial community.

Quorum sensing (QS) is a well-established mechanism by which bacteria communicate with one another by sensing and producing signaling molecules, e.g., homoserine lactone (HSL) [82]. Different bacterial taxa have the ability to generate the same type of signaling molecules, which enable either interference or cooperation with the neighboring microbes belonging to other taxa [83]. QS facilitated the movement of finger millet endophyte M6 (*Enterobacter* sp.) towards the root invading microbe *Fusarium graminearum*, and resulted in the development of biofilm on root hair to trap and prevent the entry of endophytic pathogen [84]. Terpene and HSL biosynthetic genes are enriched in microbial communities [82]. Terpenoids play roles in diverse ecological and biological functions, including chemical defense against pathogens and herbivores [85,86]. Bacterial terpenes are also involved in interkingdom signaling, as these compounds function as elicitors of profound responses in plants [87]. Microbial colonization on the ‘local side’ stimulates the microbial colonization on the ‘systemic side’ by modulating root exudation of metabolites via long-distance communication with different parts of rhizospheric system [88]. For instance, locally inoculated bacterial strains belonging to genera *Bacillus* and *Pseudomonas* trigger the production of bacteria-specific metabolites in the root exudates, which, in turn, induce the colonization of selective bacterial groups on the systemic side [89]. QS is an intercellular communication system, in which particular signal molecules including AHLs mediate bacterial gene expression and bacterial cell concentration [90]. In fact, QS system controls the production of a variety of phenotypes, many of which have been linked to pathogenesis in a variety of economically significant bacterial pathogens, such as *Pectobacterium carotovorum*, *Pseudomonas syringae*, *Dickeya solani*, *Ralstonia solanacearum*, *Erwinia amylovora*, and *Agrobacterium tumefaciens* [91]. In agriculture, interference or interrupting QS is thus an intriguing technique for preventing pathogen infections [92]. Quorum quenching (QQ), involving the enzymatic destruction of AHL signal molecules, is one of the most well-known QS-interrupting techniques [93]. For example, the QQ activity of *Pseudomonas segetis* strain P6 reduced soft rot symptoms on potato and carrot caused by *D. solani*, *P. carotovorum*, and *Pectobacterium atrosepticum* [91]. Moreover, plants are also able to disrupt the QS system by producing QS inhibitors, which either degrade QS signals or compete for signal receptors. For example, extracts from *Medicago truncatula*, cinnamon, grapefruit, and other edible plants and fruits showed QS inhibition activity against plant pathogens [94]. Bioactive molecules produced naturally by marine organisms and fungi and chemically synthesized compounds and antibodies have been reported to act as QS inhibitors. An interesting feature of QS inhibitors is that they operate at lower than minimum inhibitory concentration. Thus, pathogens do not perceive threats to their survival, and continue to grow without causing disease [95,96].

Microbial populations that can successfully colonize may not only rely on colonization signals from host plants, but also possess genetic machinery to compete with other members of the microbiome [97]. Multiple type VI secretion system (T6SS) genes, which are involved in the production of toxic proteins to kill competitor cells, have been identified in the microbial communities of wheat, cucumber [98], barley [99], and citrus [6]. Hyde1, a new family of T6SS effectors identified in pathogenic *Acidovorax* spp., was found to control the growth and proliferation of phyllospheric bacteria, revealing its involvement in inter-bacterial competition for plant colonization [44]. Therefore, an array of factors, such as fitness for competition, survival rate, ability to respond to plant-oriented signals and environmental conditions, may collectively affect the colonization process of microbial groups. However, integrated studies of modelling and synergistic interactions occurring in the plant holobiont are required to completely understand the molecular basis of complex web of microbial associations.

Recent studies have provided new insights into the role of eco-evolutionary processes, such as microbial shift between niches, selection of the fittest microbial group, ecological drift, and genetic diversification, in the microbial community assembly process [39]. Random habituation and past events have substantial impacts on the structure of microbial community in plant microbiomes. Initially, random processes trigger the colonization of rhizospheric and phyllospheric microbial communities [17,100]. Despite the robustness of primary microbial communities, plants can accommodate new species without any major change in existing microbiome structure. Deciphering the driving machinery behind the assembly, dynamics, occurrence, and sensitivity to biotic/abiotic factors is necessary to highlight the role of microbiomes in plant physiological responses to challenging environmental conditions. 

### 3.3. Plant-Pathogen Interactions 

Plant–pathogen interactions are mediated by interplays of multifaceted processes, which are facilitated by the pathogen- and plant-oriented molecules [101,102]. Molecules secreted by pathogens are the main factors, which control their successful penetration, colonization, and pathogenicity inside the host plants [103]. For instance, *Zymoseptoria tritici*, a devastating wheat pathogen, favored its infection process by suppressing immune responses of wheat plants via altering benzoxazinoids and phenylpropanoids biosynthetic pathways [104]. By contrast, plant-derived molecules are responsible for the pathogen recognition to provoke an array of defense responses within plants. The interactions between the pathogens and the plants are normally initiated in apoplast, followed by the recognition of microbial elicitors by receptors in plants [105]. These elicitors, termed as pathogen-associated molecular patterns (PAMPs), are generally recognized by membrane localized pattern recognition receptors (PRRs) in plants [102,106,107]. Bacterial flagellin, peptide surrogates, elongation factor (EF), chitin, elf18, and flg22 are common PAMPs, which are usually recognized by PRRs, i.e., chitin elicitor receptor kinase1 (CERK1), EF-Tu receptor (EFR), and flagellin-sensitive2 (FLS2), respectively. 

Recognition of microbe-derived PAMPs by plant PRRs triggers the first line of defense in host plants, termed as PAMP-triggered immunity (PTI). To overcome PTI, pathogens evolutionarily develop and deliver a large number of effector proteins into their host cells to inhibit or scavenge the PTI components [108,109]. Among these, avirulence (Avr) proteins, such as transcription activation-like effectors (TALEs) of *Xanthomonas oryzae* and S1p1 of *Magnaporthe oryzae*, are the common examples of microbial effectors that chock the defense responses of host plants [102]. As consequence, however, plants encode various disease resistance (R) proteins to recognize pathogen-associated Avr proteins [108]. Molecular interactions between host-derived R and pathogen-oriented Avr proteins activate the second line of defense, known as effector-triggered immunity (ETI). ETI is relatively faster and stronger than PTI, and usually induces localized necrosis of both pathogen and plant cells in the infected area [110,111]. PTI and ETI together constitute the innate immunity system in plants, which enable plants to recognize and resist/combat against invading pathogens. 

The fate of plant–pathogen interactions is decided by various environmental factors including cultivation conditions, genetic basis of plants and pathogens, variations in host physiological responses under biotic/abiotic climate changes, and the structure of the host-associated microbiome [17]. For example, the bacterial pathogen, *Pseudomonas fuscovaginae*, was responsible for disease incidence in highland rice plants, while the fungal pathogen, *Sarocladium oryzae*, was found to cause infection in rice plants grown at low altitudes, especially during wet season [112]. The plant microbiome, especially rhizobiome, acts as a protective shield and obstructs plant–pathogen interplays with subsequent beneficial impacts on plant health [113]. Plant-associated microbial communities hinder the pathogen-oriented activities via competing for resources, locking key nutrients (i.e., iron) and producing pathogen-killing metabolites (such as antibiotics and toxins) that ultimately eradicate the pathogens [114,115]. Furthermore, plant-beneficial microbiota also interrupt the communications between pathogens and their corresponding hosts indirectly, by inducing systemic resistance in plants (described in later sections) [116]. Recently, the indigenous plant-associated microbial communities have attracted the attentions of the scientific community, owing to their functional benefits on plant health under challenging climatic conditions [117,118].

## 4. Functions of Microbiome in Plant Health

Generally, each microcosm member possesses several valuable traits that help to regulate the physiological processes in the host plants under diverse environmental conditions. Specific traits displayed by an individual microbial group in the microbiome is of significant importance regarding plant health, and is influenced by variety of factors including microbial diversity, environmental factors, and host plant species [119,120]. The direct health-related benefits provided by the microorganisms to their host plants include nutrient acquisition, mitigation of environmental stresses, and protection from pathogens [59]. Plant microbiomes have long been studied for their role in protecting their hosts from phytopathogens, and the term “biocontrol” refers to the processes that eliminate disease-causing organisms [121]. Usually, beneficial microbes protect plants from pathogen attacks either directly (by interacting with pathogens) or indirectly (by activating the innate immune responses of the host plants). 

### 4.1. Roles in Direct Suppression of Plant Pathogens

The plant-microbiota members include neutral, pathogenic, and beneficial organisms. During their lifespan, plants not only establish beneficial associations with microbial communities, but also need to cope with the infections caused by diverse pathogenic microorganisms. Soil-borne pathogens cause adverse effects on hundreds of plant species, including economically important crops, leading to significant economic losses by reducing quality and yield [122,123,124]. The most important soil-borne fungal pathogens are *Rhizoctonia solani* [125], *Fusarium oxysporum* [126], *Verticillium* spp. [127], and *Fusarium solani* [4]. These soil-borne pathogens can survive in soil for long periods of time by forming resting structures (such as chlamydospores, melanized mycelia, oospores, cysts, and sclerotia) until they receive life signals from their corresponding host plants [59]. For example, free amino acids, phenolic compounds, and sugars in root exudates of watermelon and tomato significantly enhanced the sporulation and spore germination of *F. oxysporum* [128,129]. The infection of soil-borne pathogens usually causes root development inhibition, root rot, stunted growth, stem or collar rot, wilting, and seedling damping-off of plants [130,131], and some of the soil-borne pathogens infect a wide range of host plants rendering traditional control measures ineffective [132]. A few groups of soil-inhabiting bacteria have been known to negatively regulate the plant health. For instance, *A. tumefaciens*, the causal agent of crown gall disease [133], and *R. solanacearum*, causing bacterial wilt, are widely studied plant-damaging soil-borne bacteria [134].

Pathogens must interact with the complex microbial community of rhizosphere to develop an intense pathogenic impact on plants [135]. Pathogens negatively affect the plant health by interacting with beneficial microbiota, i.e., competing for nutrients and space, and the production of antimicrobial compounds [136]. Furthermore, pathogens also promote the colonization of other plant-harmful microbes by delivering effector proteins that cease the activities of beneficial microbes in rhizosphere community [136]. Plants and their associated microbiota are evolving simultaneously for millions of years, and this co-association of microbes and plants provides several benefits to plants including nutrient acquisition, fight against abiotic stresses, and disease suppression [111]. Host-linked communities of beneficial microbes are involved in disease suppression and nutrient mobilization in plants [137,138]. For example, *Pseudomonas* spp. can reduce the growth of plant pathogens through competition and antibiosis; however, the overall disease suppression in soil is affected by multiple factors, i.e., genetic background of both hosts and pathogens, population dynamics of pathogens, diversity and composition of plant microbiota, as well as biotic and abiotic conditions [42,139]. Although the disease suppression ability is associated with synergistic efforts of microbes rather than individual specific efforts [140,141], complete understanding of underlying interactions between potential antagonists and disease-causing phytopathogens requires further investigation. Some simple mechanisms, i.e., production of antimicrobial metabolites and volatiles in antagonistic bacteria, have been reported to be responsible for improving the efficacies of the disease-suppressive soils [142,143]. Among all mechanisms of disease suppressive soils, antibiosis (i.e., production of antimicrobial metabolites by an organism to suppress the growth and proliferation of another organism) is the most widely studied [144]. Antibiotics such as 2,4-diacetylphloroglucinol (DAPG) and phenazines (PHZ) have been well-studied, owing to their potential roles in plant disease suppression [144,145]. Several *Pseudomonas* species produced DAPG and PHZ in soils, which suppressed Fusarium wilt of flax or wheat [146]. Moreover, DAPG and pyrrolnitrin suppressed the growth of *R. solani* [147], while PHZ and pyoluteorin were widely distributed in soils and are involved in the suppression of *Thielaviopsis basicola* [148]. A rice seed endophyte, *Sphingomonas melonis*, promoted the rice panicle rot disease suppression in rice seedlings by producing anthranilic acid against *Burkholderia plantarii* [149]. Similarly, microbes with inherent potential of volatile organic compound production have been proposed as key components of disease suppressive soils [144]. Earlier studies highlighted the potential role of ammonia and hydrogen cyanide in suppressing the growth of phytopathogens [144,150,151]. Inhibition of the proliferation of pathogens in soil can be mediated by competition between plant-beneficial and -harmful microbes for survival, nutrient acquisition, and colonization [152,153].

If a pathogen successfully surpasses the rhizobiome, the so-called first line of defense against invading pathogens, and enters the plant, endophytes come into action to provide plants with an extra layer of protection. Upon pathogen entry, endophytes start recruiting microbial communities, which initiate their genetic machineries to produce defensive enzymes and metabolites against pathogens [79]. Fusarium wilt disease in various crops (e.g., tomato, lettuce, and cucumber) was suppressed when these crop plants were grown in disease suppressive soils enriched in bacterial phyla named Acinetobacteria and Firmicutes [12]. General or specific disease suppression can be achieved via modulating the composition of microbial communities by implementing different management practices, such as crop rotation and compost addition [154]. 

Genomic studies have added more knowledge about the presence of specific gene clusters that are involved in pathogen inhibitory activities. These specific gene clusters also lead towards the identification of antibiotics [80]. Plant-associated microbes are good sources of potential antagonists which can resist phytopathogens. These microbiomes interact directly with pathogens and inhibit their growth [155]. Additionally, niche overlap with microbes for resource competition is also considered a major factor in stimulating biocontrol activity [118]. 

### 4.2. Roles in Activation of Plant Immune Response

Emerging evidence has indicated that plant-associated microbiomes are engaged with plant health [156] and the beneficial features of plant-associated microbes can boost the immune responses in plants against biotic/abiotic environmental constraints [118,157]. Essentially, microbiomes help their host plants to gain resistance against pathogens via modulation of plant defense mechanisms [158,159]. The microbe-triggered immune response makes plants resilient against pathogen attack with a substantial boost in the disease combating efficiency [160,161]. Microbiomes can reinforce the defensive capabilities of plants by interrupting the plant-pathogen interactions, which subsequently improve the disease resilience in plants [89]. Bacterial antagonists belonging to *Achromobacter*, *Comamonas*, *Curtobacterium*, *Enterobacter*, *Leclercia*, *Microbacterium*, *Pantoea*, *Sphingobacterium*, and *Stenotrophomonas* genera showed tremendous biocontrol potential against *M. oryzae* and triggered the expression of defense genes, such as *OsCEBiP*, *OsCERK1*, *OsEDS1*, and *OsPAD4*, in rice seedlings against rice blast disease [162]. Similarly, root-associated *Pseudomonas* sp. EA105 and *Pantoea* sp. EA106 induced disease suppression in *M. oryzae*-challenged rice plants by triggering jasmonate- and ethylene-dependent induced systemic resistance (ISR) responses [163]. Pathogen attacks induce changes in the root exudation pattern of host plants, which can result in the colonization of specific resistance-inducing microbiota. Diverse microbial populations inhabiting the episphere and endosphere are involved in the activation of defense machinery of tomato plants against *F. oxysporum* attack by inducing cell wall fortification through the modulation of salicylic acid biosynthesis pathway [141,164]. Moreover, the root-associated microbiome induced resistance in strawberry plants against two soil-inhabiting fungal pathogens, *Verticillium dahliae* and *Macrophomina phaseolina*, in controlled field trials [165]. Similarly, a field trial revealed a positive correlation between the microbiome-triggered ability of maize plants and the suppression of a disease caused by *R. solani* [166]. Several microorganisms could induce plant immune responses under greenhouse conditions, but the majority failed in field conditions. This inconsistency is mostly linked to the lack of ability of the microorganisms to survive and colonize the rhizosphere under inappropriate environmental conditions that ultimately affects their protective qualities in field circumstances [144]. Overall, environmental conditions and soil health play important roles in the development of such beneficial plant–microbe interactions.

**Figure 3:**
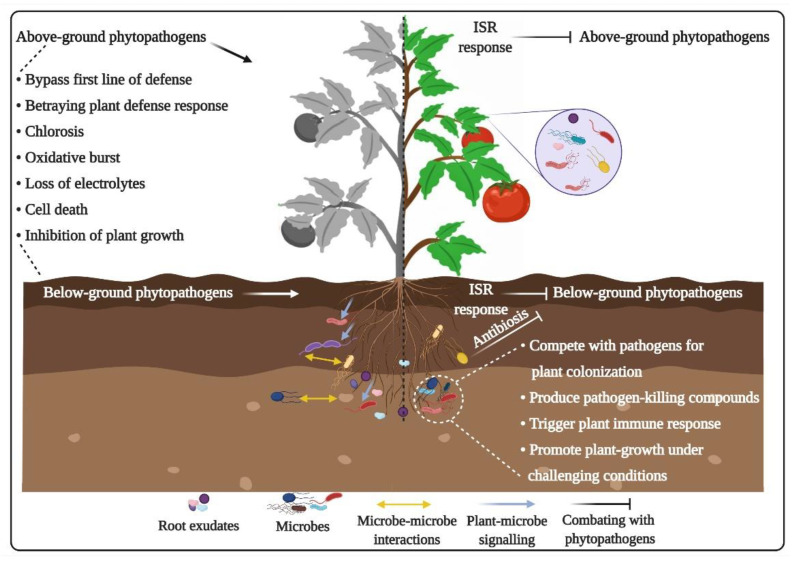
Beneficial impacts of positive plant–microbe interactions on plants. In plants, pathogen-oriented molecular patterns (i.e., PAMPs) or plant-beneficial bacteria activate induced systemic resistance (ISR). Positive interactions between plant and microbiome help plants in combating diseases and other environmental stresses, as well as boosting growth and biomass production. Beneficial plant-microbiota compete with phytopathogens for colonization, resources, and habitat, etc. Overall, beneficial microbes, through numerous mechanisms, including antibiosis, toxin production and nutrient sequestration, directly suppress the proliferation of pathogens and the symptoms of infections in plants.

Rhizobacteria conferred ISR is one form of the inducible immunity in plants, and the molecular mechanisms in ISR are conserved in different plant species [167]. Some of the components present on the cell surface of biocontrol bacteria, i.e., flagella or polysaccharide [168], can trigger ISR in plants [169]. The ISR response in plants can also be triggered when they come into direct contact with compounds secreted by beneficial bacteria, e.g., volatile 2,3-butanediol [170], DAPG, and cyclic lipopeptide surfactants [171]. A transcription factor, MYB72, is involved in the regulation of ISR in *A. thaliana* [172]. Occurrence of ISR indicates that plants might be evolved in such a way that they use associated microbes as signals for the stimulation and maturation of their immune system. Indeed, plants require microbes during the early stages of life to be in contact with soil for their survival [17]. On the other hand, ISR inducing strains regulate the plant–pathogen interactions via regulating the secretion of antimicrobial compounds by roots [137,173]. Alterations in the composition of root exudates ultimately manipulate root microbiomes and activate recruitment of plant-beneficial microbial groups in the rhizosphere [174]. Microbiome-induced resistance has been reported in different plant species for various diseases, including potato scab, sugar beet Rhizoctonia damping-off, Fusarium wilt, and wheat take-all [118,175]. Colonization of potential antagonistic bacteria conferred resistance to tomato plants against bacterial wilt pathogen *R. solanacearum* [77]. These observations indicate the possibility that the plant immune responses can be modulated by facilitating the recruitment of resistance-inducing microbes that ultimately help plants to sustainably combat pathogen attacks. Selective enrichment of microbial groups is responsible for the induction of immune responses in plants against biotic and abiotic stresses, and this induced immunity can be transferred over generations [176]. However, ISR-inducing microbes need to bypass the plant immune system to develop a symbiotic relationship with their host plants. Beneficial microbes employ mechanisms similar to pathogens which suppress activities of plant immune responses [177,178]. For example, *Rhizophagus intraradices*, an ISR-inducing arbuscular mycorrhizal (AM) fungus, suppresses plant immune responses and promotes its root colonization by producing SP7 effector [179]. Likewise, *Laccaria bicolor*, a symbiotic ectomycorrhizal fungus, successfully colonizes plant tissues by suppressing salicylic acid-mediated immune responses through MiSSP7 effector [180]. Moreover, compromised immune responses have also been reported in *A. thaliana* after colonization by *Trichoderma* [181], *Bacillus subtilis* [182], and *Pseudomonas fluorescens* WCS417r [183]. In addition to downregulating local immune responses to facilitate colonization, plant-beneficial microbes also produce elicitors/signals to activate systemic immune responses [137]. However, the detailed molecular mechanisms of mutualistic plant–microbe interactions need further investigation. The beneficial services provided by the microbiome to pathogen-challenged plants are presented in Figure 3.

## 5. Microbiome Engineering: Plausible Functional Benefits on Plant Health

**Figure 4:**
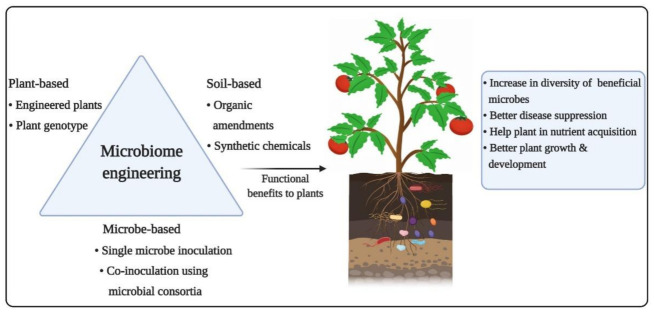
Different microbiome engineering approaches, such as cultivation of microbe-recruiting plant cultivars, inoculation of synthetic microbial communities, and conditioning of soil using suitable amendments, increase the diversity of functionally active and diverse microbial communities, resulting in improved plant health under adverse environmental conditions.

Plant microbiota is one of the primary factors responsible for the growth and development of plants under diverse environmental conditions [184]. Several emerging microbiome engineering strategies, such as soil conditioning, artificial microbial consortia, and host-dependent microbiome engineering, have been shown to strengthen these features of stress tolerance, disease resistance, and nutrient acquisition in host plants (Figure 4).

### 5.1. Traditional Soil Conditioning Using Organic and Chemical Amendments

Improvement of soil health is associated with consistent diversity of functional microbiota that will ultimately result in environmentally resilient and higher-yielding crops. Soil organic formulations can be used for supporting the growth and proliferation of functional microbial groups, and include compost, organic residues, organic wastes, biochar, and peat [11]. Biofumigation, as an organic soil conditioning, is a strategy to suppress diseases via soil fungistasis [185]. Amendment of soil with organic conditioners can enrich in positive, functionally more efficient, and interrelated species of microbes compared to supplementation with chemical fertilizers [186]. Functional characterization of positive microbial groups responding to specific organic amendments, and optimization of organic soil amendment applications for particular crop/soil type, will help to better understand the biochemistry of soil health and establish sustainable soil health. 

Plant-oriented signaling molecules, such as salicylic acid and various metabolites in root exudates, strongly affect the dynamics and composition of microbiome [156,187], suggesting that plant microbiomes can be artificially modulated using such types of microbe-stimulating chemicals in an efficient and precise manner. For example, phenolic compounds (i.e., coumarins) exuded from plant roots have an assisting role in altering the composition of root-colonizing microbes [188]. Several studies suggested the protective role of coumarins for plants against soil-borne pathogens by facilitating the growth of beneficial rhizobacteria [188,189]. In maize plants, another class of root exudates, called benzoxazinoids, has been shown to protect plants from herbivore insect attacks by favoring the recruitment and colonization of beneficial bacterial and fungal microbial groups in rhizosphere [190]. Further investigations on how different root exudates, such as malic acid, coumarins, benzoxazinoids, and camalexin, can contribute towards microbiome engineering and chemical communications between a particular signaling molecule and microbial group will help to develop host-specific biofertilizers. 

### 5.2. Microbiome Engineering Using Artificial Microbial Consortia

Like synthetic biology, the function and structure of plant microbiomes can be modulated in a more specific manner by using the approach of microbiome engineering encompassing the use of artificial microbial consortia (AMC). This approach enables us to establish AMC equipped with multiple functions relevant to plant growth and development under normal and challenging environments. Such a strategy provides the best alternative to solve numerous drawbacks associated with traditional biofertilizers, such as the inability to compete with microbes under field trials, compromised performance under local environment, and host compatibility issues [191]. The fabrication of an ideal AMC is a systematic approach involving a series of steps including the selection of microbe origin, excavating and culturing the core microbiota, identification of functionally active microbial groups, fine-tuning the microbe–microbe interactions, and the evaluation of consortium efficacy [192]. 

Numerous microbes establish complex interaction networks with other microbes in the rhizosphere, and have become a key part of the functional consortia. For example, plant growth promoting rhizobacteria (PGPR) and AM fungi can complement one another with respect to ecosystem functioning and nutrient availability [193]. Similarly, key microbial strains can also be artificially inoculated into soils to alter the structure of microbial communities [194]. The role of AMC in conferring stress tolerance to plants is well documented [172,195]. For example, co-inoculation of AM fungus *Claroideoglomus claroideum* and plant-beneficial bacterium *Pseudomonas libanensis* into sunflower rhizospheres promoted plant growth by stimulating the growth of plant-beneficial microbiota under salinity or metal stress [196]. Similarly, treating chili plant roots with a bacterial consortium including *Acinetobacter* sp., *Bacillus velezensis*, and *Bacillus amyloliquefaciens* promoted the plant growth and disease suppressive ability against soil-borne *Phytophthora capsica* [2]. In addition, *Agrobacterium* sp. modulated the bacterial community shift in rhizospheric region by promoting the growth of various PGPRs, e.g., *Brevibacterium* spp. and *Actinomycetes* spp. [197]. The *Agrobacterium*-induced microbial community shift exerted beneficial effects to bean plants by increasing overall plant biomass, antioxidants, flavonoids, potassium content, and root nodules [197,198]. Moreover, co-inoculating the rhizospheres of tomato with *Stenotrophomonas maltophilia* and *Pseudomonas stutzeri* boosted the plant growth and stimulated the production of diffusible compounds (i.e., dimethyl disulphide), which are active against the foliar pathogen *Botrytis cinerea* [199]. Two synthetic microbial communities, comprising bacterial strains with 1-aminocyclopropane-1-carboxylic acid deaminase activity, were recently constructed, and these synthetic microbial consortia showed antimicrobial potential against *F. oxysporum* f. sp. *Lycopersici* and promoted the growth of tomato plants [200]. Similarly, co-inoculation of pea plants with *Pseudomonas aeruginosa*, *Trichoderma harzianum*, and *B. subtilis* enhanced the defense response against *Sclerotinia sclerotiorum* through regulating antioxidant enzymes activities and accumulation of phenolic compounds upon pathogen attacks [201]. Application of co-cultures of *Azospirillum* sp. and *P. fluorescens* was also effective in controlling the root rot disease of cotton caused by *Rhizoctonia bataticola* [202]. Further studies unraveling the complex nexus between plant genotypes and microbial species/strains are necessary to enlighten our understanding regarding the mechanistic effect of antagonists on disease suppression. Sustainability of synthetic microbial consortia needs to be considered under field conditions, and may be achieved through continuous applications of AMC at regular intervals to stabilize microbial consortia over the generations of host plants. 

### 5.3. Host Genotype-Dependent Microbiome Engineering 

Beneficial microbes are mainly present in rhizospheres, and plant roots act as a gatekeeper to allow only beneficial microbes to enter plants as endophytes [193,199,203]. Plants also expel bacterial species into the rhizosphere, but the underlying mechanisms by which microbes (beneficial or pathogenic) exit and enter the holobiont of plants remain unknown [204,205]. Moreover, plant roots have the ability to consume associated-microbes directly as a source of nitrogen [206], indicating that microbial biomass in rhizosphere play an essential role in plant development in an unspecific manner. However, further investigation is required to understand whether plants favor specific microbes for consumption. Plant genetic machinery plays key roles in shaping and functioning of microcosms [11]. For example, *Pseudomonas simiae* WCS417r boosted the biomass production in *Arabidopsis* plants of some accessions [207]. The phyllospheric microbial diversity altered in mutant *Arabidopsis* plants defective in PTI signaling pathway and MIN7 vesicle-trafficking pathway [208]. This suggests a strong genetic connection between *Arabidopsis* plants loci (controlling plant defense and cell wall integrity) and phyllospheric bacterial diversity [209]. Mutant rice plants, deficient in jasmonate synthesis, showed a significant reduction in *Azoarcus olearius* colonization [210]. At the microbiome level, distinct plant genotypes also attract a variable range of disease suppressive and beneficial microbes, and reassemble their microbial diversity via variations in metabolites exuded from roots [77,211]. Some bacterial groups belonging to *Enterobacter* and *Kosakonia* genera are more abundant in the rhizobiomes of banana cultivars, and provide them a shield against Fusarium wilt [205]. Similarly, bean genotypes significantly affected the microbiome assembly in the rhizosphere, with only 0.7% operational taxonomic units (OTUs) in common [212]. Strong genetic correlations were detected among the diversity of epiphytic microbial population, maize plants, and their resistance to southern sheath blight pathogen *Cochliobolus heterostrophus*, and the γ-aminobutyric acid pathway was responsible for controlling the phyllospheric microbial diversity and southern sheath blight susceptibility in maize [213,214].

Selection and breeding of ‘microbe-friendly’ cultivars can provide tremendous potential for improved agricultural productivity. Knowledge on beneficial associations between plants and microbes has provided opportunities to manipulate the plant genome to attract and stabilize the functional microbes existing in the microcosm [172]. To achieve this goal, ‘designer plants’ can be genetically modified to release exudates and hormones that support the recruitment and colonization of beneficial microbiomes. Wild species or relatives may play important roles in exploring genes linked with the assembly of beneficial microbiomes [215]. For example, wild bean accessions had abundant Bacteroidetes, while modern domesticated accessions showed Proteobacteria and Actinobacteria in relatively high abundance [212]. A strong connection between host genotype and associated phylloshperic microbial diversity in different tomato accessions was detected [216]; however, host genetic variations, coupled with environmental factors, were correlated with the endophytic microbial diversity in wheat plants [217]. Thus, it is likely that microbial community shift is linked with modified plant genotypes and altered root morphology. How host genotype–microbiome crosstalk recruits beneficial microbial groups for achieving desirable traits, and how plants modulate and favor the colonization of specific microbiomes need to be investigated to devise ways to maintain functionally active and beneficial microbiomes, as well as to track the real time changes in microbial diversity under field conditions. 

## 6. Concluding Remarks and Future Perspectives

Integrated approaches of experimental biology, multi-omics, and computational biology have provided quantitative insights into plant–microbiome interactions and the underlying mechanisms. The broad survey of important crop plants and model plant species has established a list of major fungal and bacterial groups that commonly form associations with plants. However, more studies about microbial diversity are required to discover the functional consortia of microbes for agronomically important crop plants. Systematic approaches to identify core microbiota and their functions in host plants will be required to characterize microbiomes of economic and ecological importance. MWAS and GWAS have predicted key players that contain functional genes for colonizing plants, plant fitness traits, and their influence on the assembly of plant-microbiota. Although these techniques have unraveled the effects of microbiomes on plant fitness under challenging environments, large proportions of variations are not fully understood. Large-scale longitudinal studies are required to develop baseline for plant–microbiome interactions with clear consideration of host age and temporal dynamics to elucidate leftover knowledge gaps. Improved understanding about the dynamic interactions of plant–microbiome with challenging environmental conditions will give a way forward to engineer microbial consortia with robust outcomes and predicted behavior. Furthermore, coupling experimental approaches with modelling will accelerate the scientific advancement by resolving methodological and technical challenges associated with the plant–microbiome world. Integrative approaches, combining the knowledge from different scientific disciplines, will help to engineer and boost the activities of complex microbial consortia in a consistent and precise manner. Improved knowledge about the dynamics of plant–microbiome–environment interplay will pave the way for the deployment of engineered microbial consortia for sustainable and improved plant production under a continuously fluctuating environment.

## Figures and Tables

**Table 1:** Summary of some root exudates involved in plant–microbe crosstalk with subsequent impact on plant microbe symbiosis.

Root Exudates	Microbial Receptors ^1^	Microbes	Plant Species	Effects	References
Alanine	CtaA, CtaB, CtaC and CtaD	*Pseudomonas protegens* CHA0	Tobacco	Suppresses root disease	[58]
Arginine	McpA	*Azospirillum caulinodans*	*Sesbania rostrata*	Regulates root colonization and flagella synthesis	[59]
Chitinase	McpU	*Pseudomonas* sp. RP2	*Arachis hypogaea*	Increases immunity	[55]
Choline	McpX	*Sinorhizobium meliloti*	Alfalfa	Facilitates nitrogen-fixation in root nodules	[60]
Citric acid	McpU	*Sinorhizobium meliloti*	Alfalfa	Regulates root colonization	[61]
Citric acid	McpU	*Bacillus subtilis*	*Arabidopsis thaliana*	Enhances root binding of *Bacillus subtilis*	[62]
Ethylene	ETR1	*Azospirillum brasilense*	Wheat	Modulates plant morphology	[63]
Malic acid	McpU	*Bacillus subtilis*	Rice	Improves nutrient assimilation and pH regulation	[64]
Malic acid	McpA	*Pseudomonas fluorescens*	Tomato	Improves plant growth and nutrient acquisition	[65]
Methyl-glucoside	McpA	*Bacillus amyloliquefaciens*	Cucumber	Modulates chemotaxis mobility and enhances immunity	[48]
Nicotine	McpU	*Pseudomonas aeruginosa*	Tobacco	Increases biocontrol efficiency against bacterial wilt	[66]
Oxalic acid	TlpA1	*Azospirillum caulinodan*	*Sesbania rostrata*	Increases plant growth	[67]
Proline	IcpB	*Pseudomonas aeruginosa*	Cucumber	Shows antifungal activity against *Fusarium oxysporum*	[68]
Proline	McpB	*Bacillus velezensis*	Maize	Regulates swarming motility and biofilm formation	[69]
Proline	McpU	*Sinorhizobium meliloti*	Alfalfa	Directs flagellar motor rotation and root colonization	[54]
Succinic acid	TlpA1	*Azospirillum brasilense*	Wheat	Increases plant growth, root volume, and crop yield	[41]
Succinic acid	TlpA1	*Bacillus velezensis*	*Brachypodium distachyon*	Regulates biofilm formation	[51]
Tryptophan	IcpB	*Azorhizobium caulinodans*	*Sesbania rostrata*	Modulates nodulation and nitrogen fixation	[70]
Tryptophan	IcpB	*Bacillus cereus*	Tomato	Reduces the damage of *Medoidogyne incognita*	[71]

^1^ CtaA, CtaB, CtaC, and CtaD are the chemoreceptors for amino acids; McpA, McpU, and McpX are chemotaxis sensory proteins that detect chemotactic ligands; ETR1 is a membrane-localized histidine kinase chemoattractant receptor for ethylene; TlpA1 is a transmembrane chemoreceptor that responds to various organic acids, glycerol, and proline; and IcpB is a heme-binding soluble chemotaxis sensory protein that senses organic acids.

## Data Availability

Not applicable.

## Abbreviations

AHLs, N-acylhomoserine lactones; AM, arbuscular mycorrhizal; AMC, artificial microbial consortia; Avr, avirulence; BGCs, biosynthetic gene clusters; CERK1, chitin elicitor receptor kinase1; DAPG, 2,4-diacetylphloroglucinol; EF, elongation factor; EFR, EF-Tu receptor; ETI, effector-triggered immunity; FLS2, flagellin-sensitive2; HSL, homoserine lactone; ISR, induced systemic resistance; M/GWAS, metagenome/genome-wide association studies; NRPSs, non-ribosomal peptide synthetases; PAMPs, pathogen-associated molecular patterns, PGPR, plant growth promoting rhizobacteria; PHZ, phenazines; PKSs, polyketide synthases; PRRs, pattern recognition receptors; PTI, PAMP-triggered immunity; QQ, quorum quenching; QS, quorum sensing; TALEs, transcription activation-like effectors; T6SS, type VI secretion system; *Xoo*, *Xanthomonas oryzae* pv. *oryzae*.
